# Submandibular Gland Pathogenesis Following SARS-CoV-2 Infection and Implications for Xerostomia

**DOI:** 10.3390/ijms25136820

**Published:** 2024-06-21

**Authors:** Estela Sasso-Cerri, Vitor Dallacqua Martinelli, Salmo Azambuja de Oliveira, André Acácio Souza da Silva, Juliana Cerini Grassi de Moraes, Paulo Sérgio Cerri

**Affiliations:** 1 Laboratory of Histology and Embryology, Department of Morphology, Genetics, Orthodontics and Pediatric Dentistry, Dental School–São Paulo State University (UNESP), Araraquara 14801-903, Brazil; vitor.dallacqua@unesp.br (V.D.M.); juliana.cerini@unesp.br (J.C.G.d.M.); 2 Department of Morphology and Genetics, Federal University of São Paulo, São Paulo 04023-900, Brazil; salmoazambujadeoliveira@gmail.com (S.A.d.O.); andre.acacio@unesp.br (A.A.S.d.S.)

**Keywords:** salivary gland, acinar cell, sialadenosis, Sjögren’s syndrome, mucin, telocytes, myoepithelial cell, DMV, transmission electron microscopy, immunofluorescence

## Abstract

Although SARS-CoV-2 induces mucin hypersecretion in the respiratory tract, hyposalivation/xerostomia has been reported by COVID-19 patients. We evaluate the submandibular gland (SMGs) pathogenesis in SARS-CoV-2-infected K18-hACE2 mice, focusing on the impact of infection on the mucin production and structural integrity of acini, ductal system, myoepithelial cells (MECs) and telocytes. The spike protein, the nucleocapsid protein, hACE2, actin, EGF, TNF-α and IL-1β were detected by immunofluorescence, and the *Egfr* and *Muc5b* expression was evaluated. In the infected animals, significant acinar hypertrophy was observed in contrast to ductal atrophy. Nucleocapsid proteins and/or viral particles were detected in the SMG cells, mainly in the nuclear membrane-derived vesicles, confirming the nuclear role in the viral formation. The acinar cells showed intense TNF-α and IL-1β immunoexpression, and the EGF-EGFR signaling increased, together with *Muc5b* upregulation. This finding explains mucin hypersecretion and acinar hypertrophy, which compress the ducts. Dying MECs and actin reduction were also observed, indicating failure of contraction and acinar support, favoring acinar hypertrophy. Viral assembly was found in the dying telocytes, pointing to these intercommunicating cells as viral transmitters in SMGs. Therefore, EGF-EGFR-induced mucin hypersecretion was triggered by SARS-CoV-2 in acinar cells, likely mediated by cytokines. The damage to telocytes and MECs may have favored the acinar hypertrophy, leading to ductal obstruction, explaining xerostomia in COVID-19 patients. Thus, acinar cells, telocytes and MECs may be viral targets, which favor replication and cell-to-cell viral transmission in the SMG, corroborating the high viral load in saliva of infected individuals.

## 1. Introduction

According to the World Health Organization, SARS-CoV-2 is an airborne virus transmitted by infected droplets and aerosols, either by symptomatic or by asymptomatic individuals [1]. Viral RNA has been detected in the saliva of 87–100% of COVID-19 patients [2], and although most of infected individuals (±80%) are asymptomatic, they may spread the virus [3]. The viral receptor angiotensin-converting enzyme 2 (ACE2) has been detected in the oral mucosa and the salivary glands [3], and the expression of ACE2 in minor salivary glands is higher than in the lungs [4]. In COVID-19 patients, SARS-CoV-2 has been detected in the salivary glands [3,4,5], and saliva samples are more sensitive to SARS-CoV-2 RNA detection than the nasopharyngeal smear samples in the infected patients [6,7]. Thus, these findings have identified the salivary glands as potential targets and major sources of SARS-CoV-2 [8].

Saliva is a specific and complex fluid composed of numerous proteins, such as acid and basic proline-rich proteins, α-amylases, cystatins, histatins, statherin and host defense peptides, which have antibacterial, antifungal and antiviral properties. Mucins are highly glycosylated proteins, produced mainly by the mucous acinar cells of the submandibular, sublingual and minor salivary glands [9]. These proteins may be of two types: (1) membrane-bound mucins, named transmembrane mucins, located on the apical surface of the epithelial cells in the digestive and respiratory systems, as well as in the liver, pancreas, kidney, gall bladder, salivary glands, lacrimal glands, and eyes [10]; (2) gel-forming mucins, secreted, for example, by goblet cells and salivary gland cells. MUC5AC is a gel-forming mucin highly expressed in the stomach and the lungs, whereas MUC5B is also expressed in the lungs [10] and by the mucous cells of the submandibular, sublingual, palatal and labial salivary glands, being the main secreted mucin in the oral cavity. MUC5B influences the composition of the oral microbiota, avoiding the harmful effects of opportunistic pathogens, such as *C. albicans*; for this reason, these pathogens remain in the oral cavity without causing oral diseases [9].

Most of the saliva is produced by the submandibular gland, composed of acinar secretory units, which are predominantly of the mucous type, and a ductal system. Unlike humans, whose submandibular glands’ (SMGs) ducal system contains three segments, the SMGs in rodents contain four segments: the intercalated duct, the granular convoluted tubule (GCT), the striated duct and the excretory duct. The GCT is a specific segment between the intercalated and the striated ducts [11], found more frequently in male than in female rodents due to its responsiveness to androgens [12]. These tubules are composed of granular epithelial cells whose granules contain several growth factors, including epidermal growth factor (EGF) [11,12], a mitogenic factor essential for cell growth, differentiation and proliferation, favoring tissue repair [13,14]. Moreover, the binding of EGF to its receptor (EGFR) triggers anti-apoptotic pathways [15,16].

The saliva secretion and release through the ducts into the oral cavity depends on the contractile function of myoepithelial cells (MECs). These stellate and flattened cells, whose cytoplasm is filled with myosin and actin filaments, contain long processes that surround the acini and tubules/ducts. Under cholinergic stimulation, MECs contract, resulting in the compression of glandular cells and the release of saliva [17,18]. Damage to myofilaments or lack of MECs impairs the excretory function of salivary glands, as demonstrated in some salivary gland diseases, such as sialadenosis (or sialosis) [19] and Sjögren’s syndrome (SS) [17], resulting in hyposalivation and xerostomia due to excretory dysfunction.

The connective tissue stroma of the salivary glands is composed of blood vessels, fibroblasts, macrophages, mast cells and telocytes. Primarily described by Popescu and Faussone-Pellegrini [20], telocytes (also known as “Interstitial Cajal-like cells”) have been identified in the stroma of several organs, such as the heart [20], lungs [21], prostate [22], pancreas [20] and salivary glands [23,24,25]. Telocytes’ morphology, function and genic expression differ significantly from fibroblast and mesenchymal cells [26]. These cells contain long processes called telopodes, which have thin (podomer) and dilated (podoms) portions and a dichotomous branching pattern [20]. In the parotid gland, telocytes interact with acini, ducts, MECs and blood vessels, maintaining glandular homeostasis [23]. In the lungs, the long telopodes are in contact with each other and with other cell types, forming an intricate network, which provides not only mechanical support but also the intercellular signaling, maintaining tissue homeostasis and favoring regeneration, immune regulation or surveillance [27]. Moreover, telocytes release extracellular vesicles, which provide an intercellular communication through the transport of signaling molecules [26]. Thus, damage to this cell type may trigger structural and functional disorders in tissues and organs.

In the autopsied lungs of COVID-19 patients, the mucus accumulation is caused by *Muc5b* overexpression, induced by SARS-CoV-2 [28]. Studies have demonstrated an intrinsic correlation between mucin hypersecretion and EGF-EGFR signaling in the respiratory cells during airway diseases, as well as in the stomach epithelial cells [29,30]. An increased number of goblet cells and AB-PAS-positive mucin has been associated with *Muc5ac* gene overexpression through EGF control [30,31]. In human bronchial epithelial cell cultures infected with SARS-CoV-2, a high expression of EGFR ligands and pro-inflammatory cytokines (IL-1β and IL-1α) has been correlated to mucin gene (*Muc5b*) overexpression. This mucin hypersecretion seems to be mediated by EGFR and IL-1R-dependent pathways [28]. In addition to IL-1, TNF-α is also related to EGF-EGFR-induced mucin secretory activity [30]. This cytokine plays an important role in the immune response against SARS-CoV-2 infection [32] and induces *Egfr* overexpression in the respiratory tract. Thus, the binding of EGF to EGFR activates the ERK signaling pathway, leading to *Muc5ac* overexpression [33].

The salivary glands are complex organs that produce a specialized fluid (saliva), whose quantity and quality are crucial for the maintenance of oral health [34]. Thus, salivary hypofunction and/or hyposalivation causes a decrease in MUC5B levels and other proteins, causing dysbiosis and oral diseases [9]. During the pandemic, numerous COVID-19 cases presented oral manifestations, including dry mouth (xerostomia) due to hyposalivation [35,36], and long-term saliva secretory dysfunction persists after recovery from COVID-19 [37]. However, since COVID-19 autopsied lungs have demonstrated a high concentration of mucus and *Muc5β* overexpression, the hyposalivation and xerostomia in the patients is intriguing. As mucin hypersecretion is triggered by cytokines [28], the submandibular gland pathogenesis in response to SARS-CoV-2 infection was evaluated in K18-hACE2 transgenic mice, focusing on the immunolocalization of IL-1β and TNF-α in acinar and ductal cells, and possible EGFR-dependent mucin secretion. In an attempt to better understand why the salivary gland is susceptible to the infection, the structural integrity of acinar cells, ductal cells, MECs and telocytes was also analyzed under light and electron microscopy.

## 2. Results

### 2.1. hACE2, Spike, Nucleocapsid and Pro-Inflammatory Cytokines in the Infected SMGs

**Figure 1:**
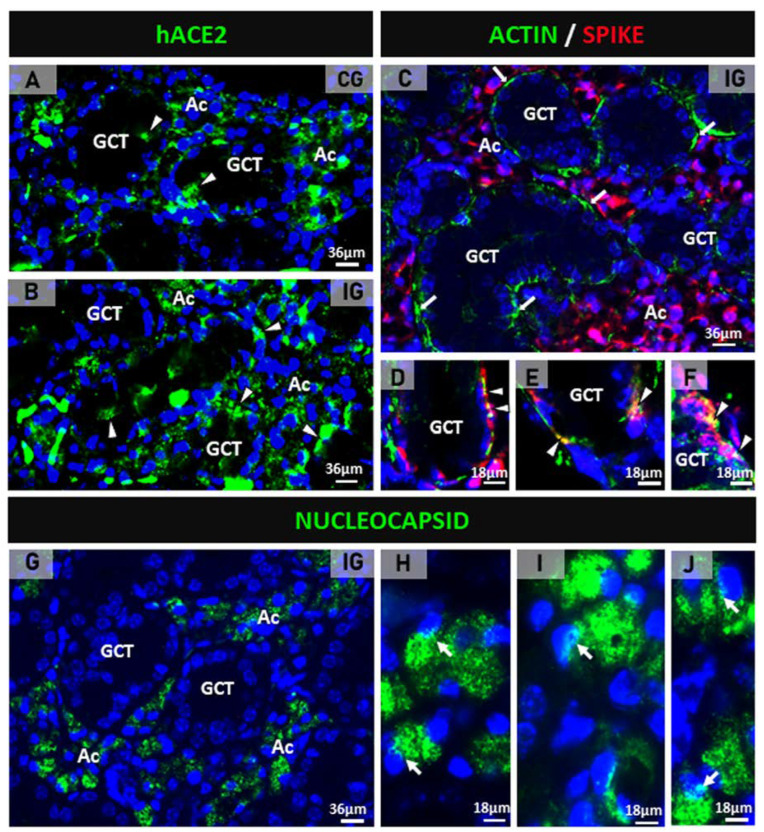
(**A**–**J**) Photomicrographs of submandibular gland sections subjected to immunofluorescence for detection of hACE2 (**A**,**B**), actin + spike double immunofluorescence (**C**–**F**) and nucleocapsid protein (**G**–**J**). In (**A**,**B**), hACE2 is observed mainly in the acini (Ac) and some punctual immunoexpression in the GCTs (arrowheads). In (**C**), the GCTs are surrounded by discontinuous actin immunoexpression (green), and an evident spike immunolabeling is observed in the acinar cells (Ac). In (**D**–**F**), co-localization of actin and spike (yellow) is noted in the myoepithelial cells (arrowheads) surrounding GCTs. In (**G**), nucleocapsid protein is observed in the acinar cells (Ac). In (**H**–**J**), nucleocapsid immunolabeling is observed filling the cytoplasm of acinar cells; note immunofluorescent reaction in close contact or within the nucleus (arrows).

Either in the CG or the IG, hACE2 immunolabeling was detected in the acini and the GCTs of the SMGs of the transgenic mice (Figure 1A,B). In the IG, the spike/actin double immunofluorescence showed discontinuous actin immunolabeling (in green) around the acini and GCTs, confirming MECs immunolabeling. Moreover, an evident spike immunolabeling was observed in the acinar cells (Figure 1C). Under high magnification, a co-localization of actin/spike (in yellow) was observed in the MECs (Figure 1D–F). In addition to the spike protein, the nucleocapsid protein was also detected in the acinar cells. Immunofluorescent masses of this protein were observed filling the cytoplasm, as well as next or inside the nucleus (Figure 1G–J). In the glandular sections used as negative controls, no immunoreaction was found (data not illustrated).

**Figure 2:**
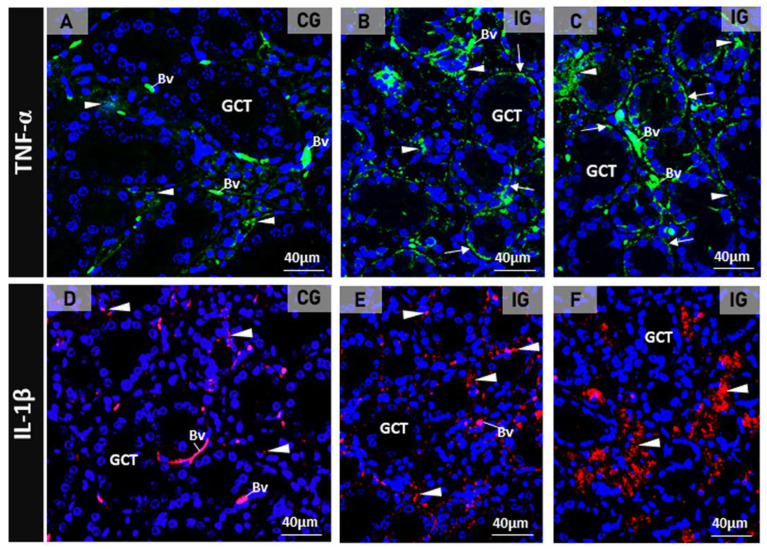
(**A**–**F**) Photomicrographs of SMGs showing immunofluorescence reactions for detection of TNF-α (**A**–**C**) and IL-1 β (**D**–**F**). In (**A**), a subtle TNF-α immunolabeling is observed in the acini (arrowheads) whereas in (**B**,**C**), a strong immunoreaction is observed in the acini (arrowheads) and basal portion of GCTs (arrows). In (**D**), the acinar IL-1β immunoreaction is weak (arrowheads) when compared to the strong acinar immunoreaction observed in E and F (arrowheads). Unspecific labelled erythrocytes are normally observed in the blood vessels (Bv).

The immunofluorescence for the detection of TNF-α and IL-1β in the SMGs of the control animals showed subtle immunolabelling in the SMG cells (Figure 2A,D). However, a strong TNF-α and IL-1β immunoreactions were observed in the acinar and/or GCT cells in the infected animals (Figure 2B,C,E,F).

### 2.2. SARS-CoV-2 Induces Acinar Hypertrophy, GCTs Atrophy and Muc5b and Egfr Upregulation

**Figure 3:**
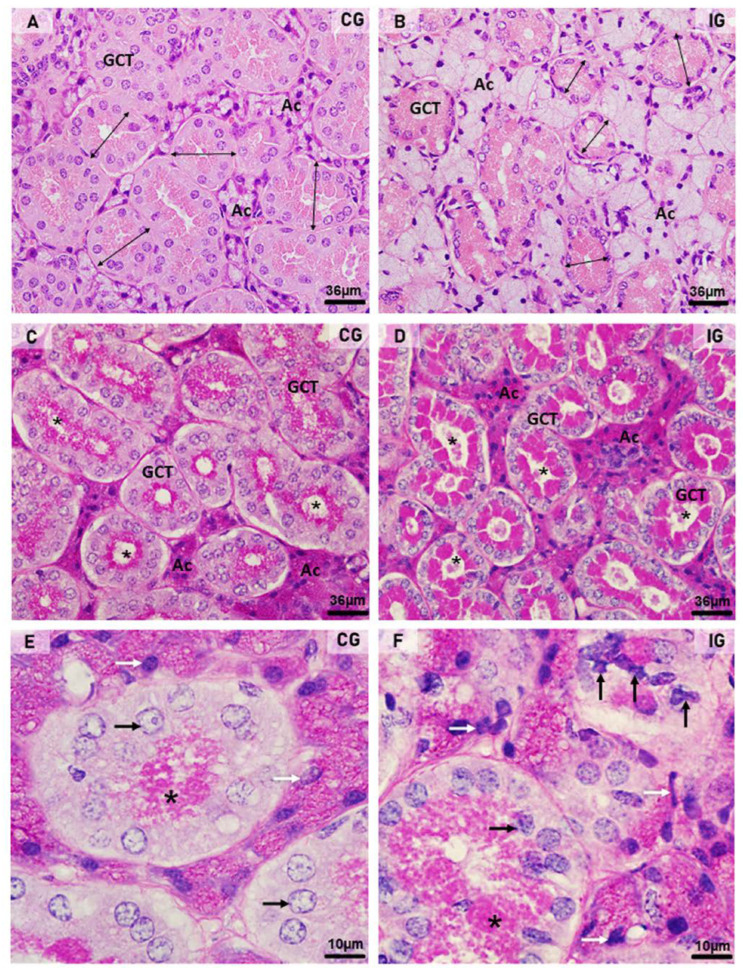
(**A**–**F**) Photomicrographs of submandibular gland sections stained by HE (**A**,**B**) and PAS method (**C**–**F**). In (**A**,**B**), acini (Ac) and GCTs are observed. Note that in (**B**) (IG), the acinar area is increased and the GCTs diameter reduced (double headed arrows) when compared to CG. In (**C**,**D**), PAS-positive secretory granules (magenta) are observed in the GCTs; however, in IG, an intense PAS staining is filling almost all the cytoplasm in comparison to the normal apical staining pattern observed in CG. Note that most GCTs show a PAS-positive secretory content in the lumen (asterisks) in comparison to CG. Ac (acini). In (**E**,**F**), GCTs show PAS-positive granules in the apical portion ((**E**); asterisk) and filling almost all the cytoplasm ((**F**); asterisk). In (**F**) (IG), either the acinar (white arrows) or GCTs (black arrows) cells show irregular nuclei with strongly basophilic condensed chromatin in comparison to CG.

**Figure 4:**
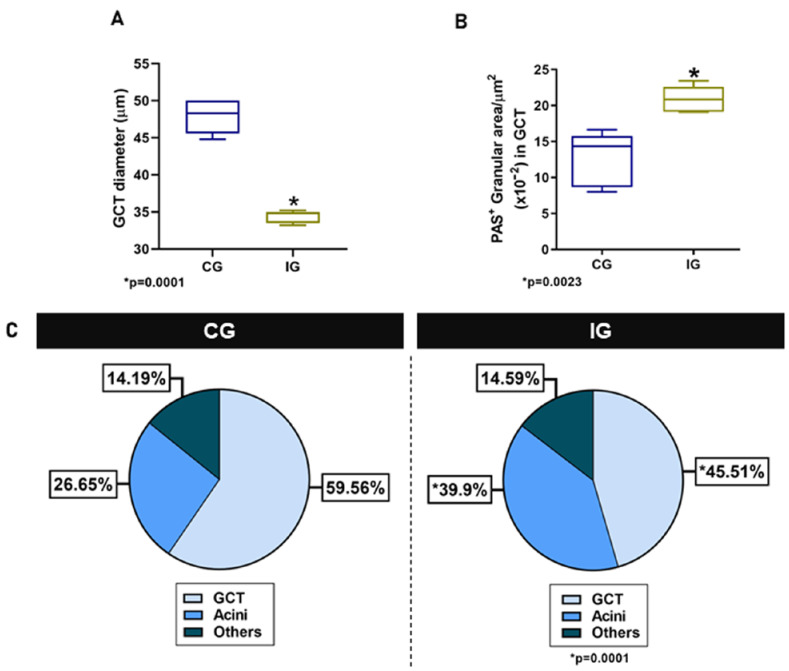
(**A**,**B**) Diameter and PAS-positive granular area in GCTs of animals from the CG and IG. (**C**) Volume density (Vv) of GCTs and acini in the animals from the CG and IG.

In the HE-stained sections of the animals from the CG, the glandular histoarchitecture was normal, showing several GCTs intermingled with acini. Conversely, in the IG, the acini were larger than in the CG, and the GCTs decreased in comparison to the CG (Figure 3A,B). These findings were confirmed by the morphometric analyses, since the GCT’s diameter and Vv decreased significantly (Figure 4A,C), whereas the acini Vv increased in comparison with the CG (Figure 4C). It is important to emphasize that in the IG, cells with an irregular nucleus and strongly basophilic chromatin, suggesting apoptosis, were usually found in both acini and GCTs (Figure 3F).

Regarding the PAS reaction, PAS-stained secretory granules were normally concentrated in the apical portion of GCT cells in the CG, whereas in the IG, the PAS-positive granules were filling almost all the cytoplasm (Figure 3C–F). Moreover, in the IG, the lumen of the GCTs was usually filled with a strongly PAS-positive secretory content (Figure 3D). The cytoplasmic area occupied by the PAS-positive granules increased significantly in the IG (Figure 4B).

**Figure 5:**
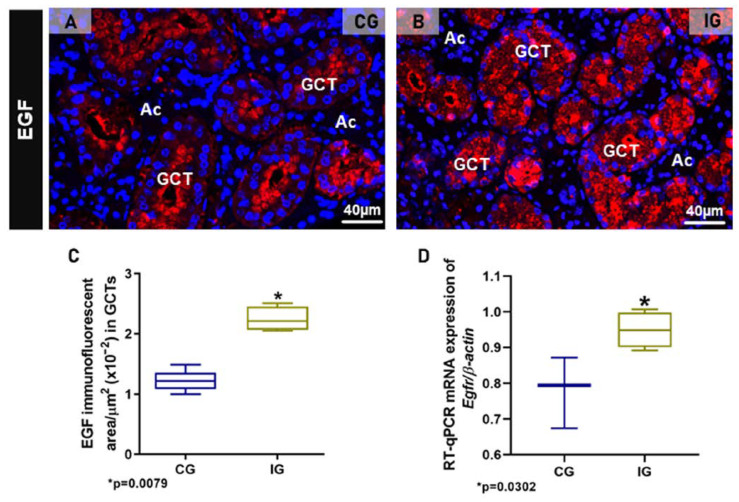
(**A**,**B**) Photomicrographs of SMGs showing immunofluorescence reactions for detection of EGF. In (**A**), an evident EGF immunoreaction is observed in the apical portion of GCTs whereas, in (**B**), a granular immunofluorescence is filling almost all the cytoplasm of tubular cells. (**C**) Immunofluorescent area of EGF in the SMGs of animals from the CG and IG. (**D**) *Egfr* mRNA levels in the SMG of animals from the CG and IG.

Either in CG or IG, the GCT granules showed also a strong EGF immunolabelling, confirming the presence of this factor in these specific salivary granules. However, whereas in CG, an evident EGF immunoreaction was noted in the apical portion of GCT cells, in IG, the EGF immunofluorescence was spread throughout the cytoplasm (Figure 5A,B). The EGF immunofluorescent area increased significantly in the GCTs of IG (Figure 5C). Moreover, a significant *Egfr* overexpression was observed in the SMGs of IG in comparison to CG (Figure 5D).

**Figure 6:**
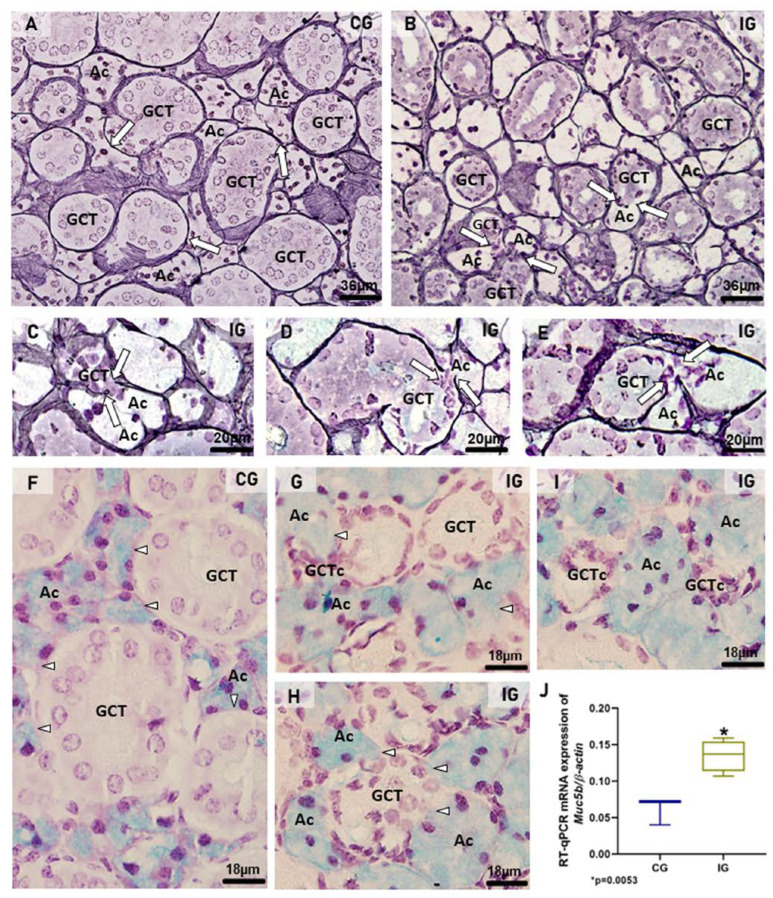
(**A**–**I**) Photomicrographs of sections of SMGs stained by silver impregnation (**A**–**E**) and AB (**F**–**I**). In (**A**), acini (Ac) and GCTs are surrounded by evident basement membrane in black (arrows). In IG (**B**–**E**), the basement membrane is discontinuous in some points where acinus (Ac) and GCT are interconnected (arrows), indicating fusion between these structures. In (**F**), AB-positive mucin is evident in the acini (Ac). The acinar–GCT interface is well delimited (arrowheads). In (**G**,**H**), the GCTs are compressed by the large AB-positive acini (Ac), and the AB-positive mucin seems to be invading the juxtaposed tubular cells (arrowheads). In (**G**,**I**), clusters of GCT cells (GCTc) are enclosed by the acinar cells (Ac). (**J**) A significant increase in *Muc5b* mRNA expression is observed in the IG in comparison to the CG.

To evaluate the acini-GCTs interface integrity, the silver impregnation for the identification of basement membrane surrounding the acini and GCTs was performed. In CG, the acini and GCTs were normally surrounded by an evident silver-stained basement membrane (Figure 6A). However, in IG, the basement membrane was discontinuous, indicating fusion and interconnection between the acini and the GCTs (Figure 6B–E). Moreover, the AB staining for mucin detection showed an evident delimitation of mucin within the acinar cells of CG (Figure 6F) whereas in IG, the large AB-positive acini were compressing the GCTs and enclosing clusters of damaged GCT cells (Figure 6G,I). Moreover, in some hypertrophied acinus-GCT interfaces, AB-positive mucin appeared to be invading the juxtaposed GCT cells (Figure 6G–I).

In the SMGs of the IG, the mRNA expression of *Muc5b* was significantly higher than that in the CG (Figure 6J).

### 2.3. Ultrastructural Features Confirm Infection, Acinar Hypertrophy and GCT Compression

**Figure 7:**
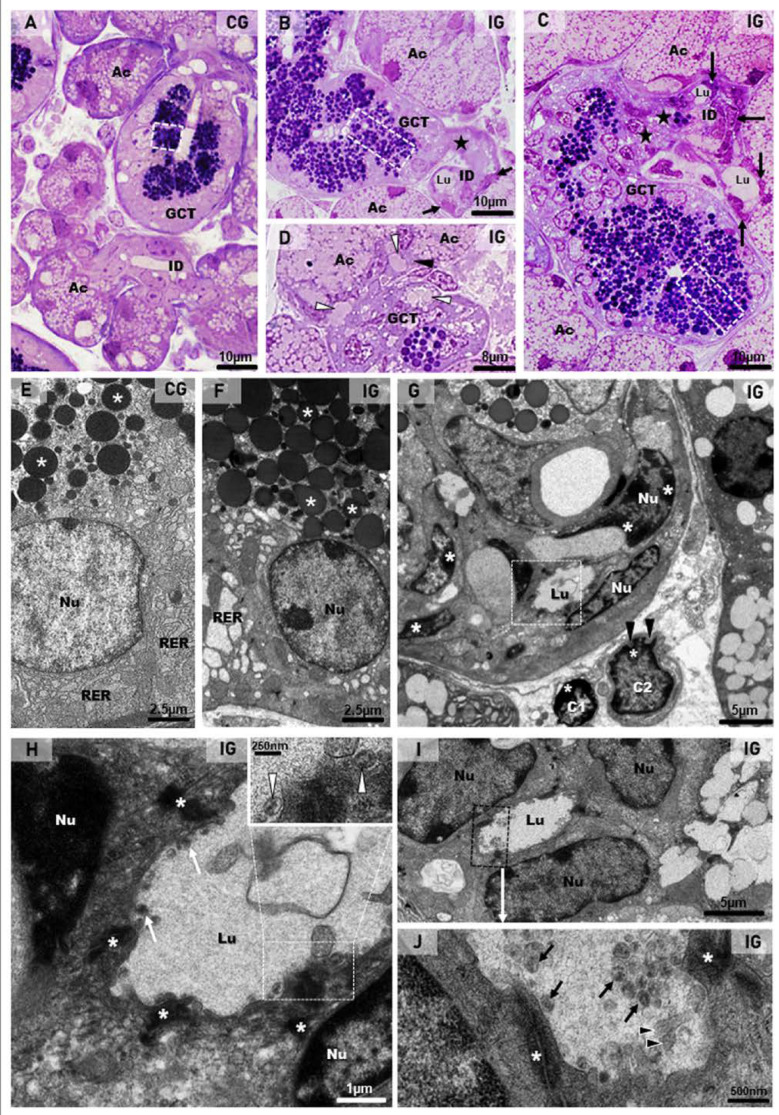
(**A**–**D**) Photomicrographs of semithin sections of SMGs stained by toluidine blue. In (**A**), the normal acini show mucin granules (Ac) and GCT contains apical granules (white box). An intercalated duct is also observed with typical lumen (ID). In (**B**,**C**) (IG), the acinar cells are larger and filled with numerous mucin granules (Ac) in comparison to the CG. In GCTs, the granules are filling almost all the cytoplasm (white boxes). Narrow and compressed portions of GCT (stars) are continuous with intercalated ducts (ID), whose cells are compacted, showing flattened and irregular nuclei (arrows). Lumen (Lu). In (**D**), a GCT portion in close contact with acini (Ac) shows mucin granules, apparently inside GCT cells’ cytoplasm (white arrowheads); some of these cells are intermingled with the acinar cells (black arrowhead). (**E**–**J**) Electron micrographs of portions of GCTs and intercalated ducts (ID). In (**E**) (CG), the GCT cell shows rough endoplasmic reticulum (RER) and numerous homogeneous spherical secretory granules (asterisks) in the apical portion. In (**F**) (IG), a high granular density is noted in the cytoplasm; some granules show irregular shape (asterisks). The RER cisternae are larger and more electron-lucent than in the CG. Nu (nucleus). In (**G**) (similar to Figure 5C), a damaged and compressed GCT portion with typical granules is in contact with an intercalated duct (ID), whose cells show flattened nuclei (Nu) with electron-dense masses of chromatin (asterisks), indicating cell death. In the stroma, note two dying cells (C1 and C2) with the nuclei showing a peripheral condensed chromatin (asterisks) and irregular outline (black arrowheads). In (**H**) (high magnification of the intercalated duct in (**G**); white box), the apical portions of epithelial cells are attached by desmosomes (asterisks) and delimiting the lumen (Lu), in which viral particles (white arrows) are observed. The viral particles are surrounded by a membrane and contain nucleocapsid proteins (inset; white arrowheads). Nu (nucleus). In (**I**), intercalated duct cells (Nu) are delimiting the lumen (Lu). In (**J**) (high magnification of (**I**); black box), viral particles are seen in the lumen (black arrows). Some microvilli of the ductal cells containing actin filaments (arrowheads) are protruding towards the lumen. Desmosomes (asterisks) are also observed.

In the submandibular gland semithin sections of the CG, the secretory granules of the GCT cells were normally restricted to the apical portion (Figure 7A). In contrast, the GCT cells in the IG showed numerous secretory granules, spread from the basal to the apical cytoplasmic portion. Some damaged GCTs showed narrow and compressed portions in continuity with the intercalated ducts (ID), which showed compacted cells with flattened and irregular nucleus (Figure 7B,C). In some portions of the acinar-GCT interface, some GCT cells were intermingled with the acinar cells and showed mucin accumulation in the cytoplasm (Figure 7D). Under TEM, the GCT cells containing typical apical granules showed a rough endoplasmic reticulum (RER) filling the cytoplasm (Figure 7E). However, in the IG, the GCT cells showed large and electron-lucent rough endoplasmic reticulum (RER) cisternae. The electron-opaque secretory granules were filling almost all the cytoplasm; some of them showed an irregular shape (Figure 7F). Portions of damaged and compressed GCTs, due to acinar hypertrophy, were also observed. These tubules were continuous with the intercalated ducts, whose cells showed flattened nucleus with masses of condensed chromatin, indicating cell death. Dying epithelial cells were also observed in the connective tissue (Figure 7G). In the intercalated ducts, enveloped viral particles containing nucleocapsid proteins were found in the lumen (Figure 7H–J). 

**Figure 8:**
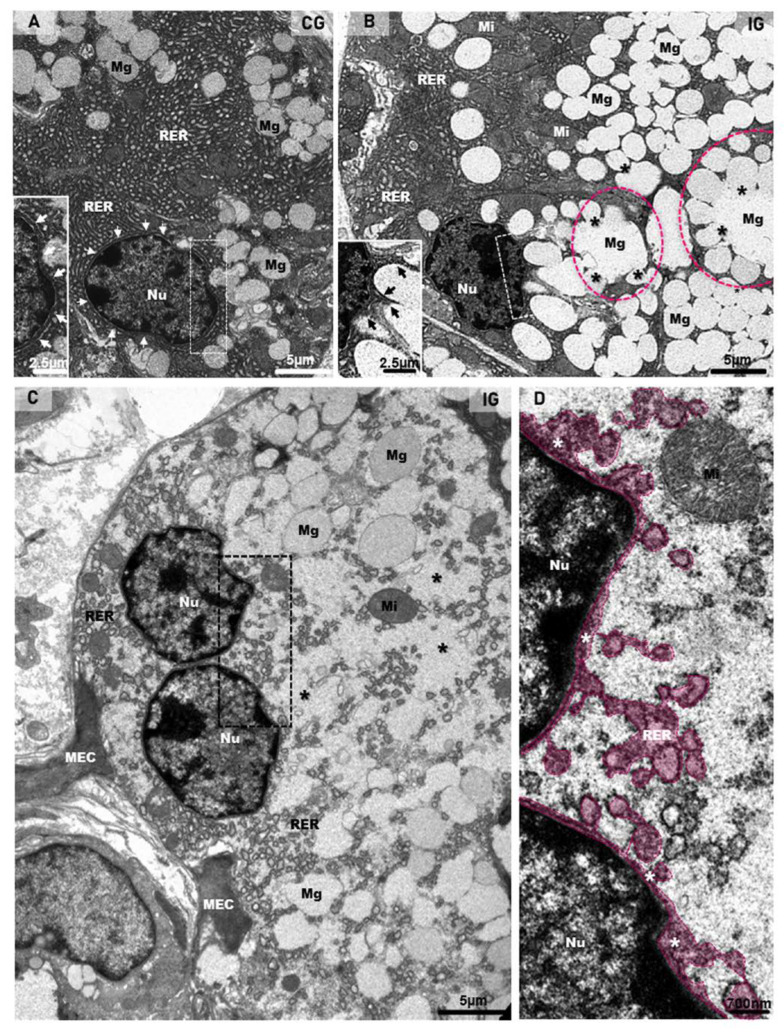
(**A**–**D**) Electron micrographs of acini of submandibular gland sections of animals from the CG and IG. In (**A**), note the organized rough endoplasmic reticulum cisternae (RER) filling almost all the cytoplasm, and mucus granules (Mg) intermingled with the RER. The intact nuclear membrane is surrounded by typical circular RER cisternae (white arrows and inset). In (**B**), numerous mucus secretory granules (Mg) are filling almost all the cytoplasm; some of them are fusing with each other (asterisks), forming large mucus granules (pink circles). The remaining cytoplasm is filled with rough endoplasmic reticulum (RER) and mitochondria (Mi). Note that the nucleus (Nu), differently from the CG (**A**), shows an irregular nuclear membrane forming protrusions towards the cytoplasm (inset; black arrows). In (**C**), a binuclear acinar cell (Nu) shows dilated rough endoplasmic reticulum (RER) cisternae intermingling with large mucus secretory granules (Mg). Irregular masses of mucus secretion (asterisks), derived from the granules’ fusion, are spread through the cytoplasm. Portions of myoepithelial cells (MEC). In (**D**) (high magnification of delimited portion of (**C**)), RER cisternae-like structures (in pink) seem to be interconnected with a dilation of the nuclear intermembrane space (white asterisks). Nu (nucleus); Mi (mitochondria).

The ultrastructural features of the acinar cells in the CG showed a typical nucleus, with intact nuclear membrane surrounded by concentric layers of RER cisternae. The cytoplasm, filled with RER cisternae and mitochondria, showed mucus granules in the apical and basal portions (Figure 8A). However, in all samples of the IG, the large acinar cells were almost completely filled with mucus granules, and several of them were fusing to each other. The mucin-congested cytoplasm showed some RER cisternae restricted to the basal portion or sparse and compressed among the large mucin granules. Differently from the CG, the nucleus showed nuclear intermembrane space dilations (similar to the RER cisternae) protruding towards the cytoplasm (Figure 8B). In the hypertrophied acinar cells, the nuclear membrane dilations were forming blebs/vesicles towards the cytoplasm, which was filled with irregular masses of mucin, derived from the fusion of mucin granules (Figure 8C,D).

**Figure 9:**
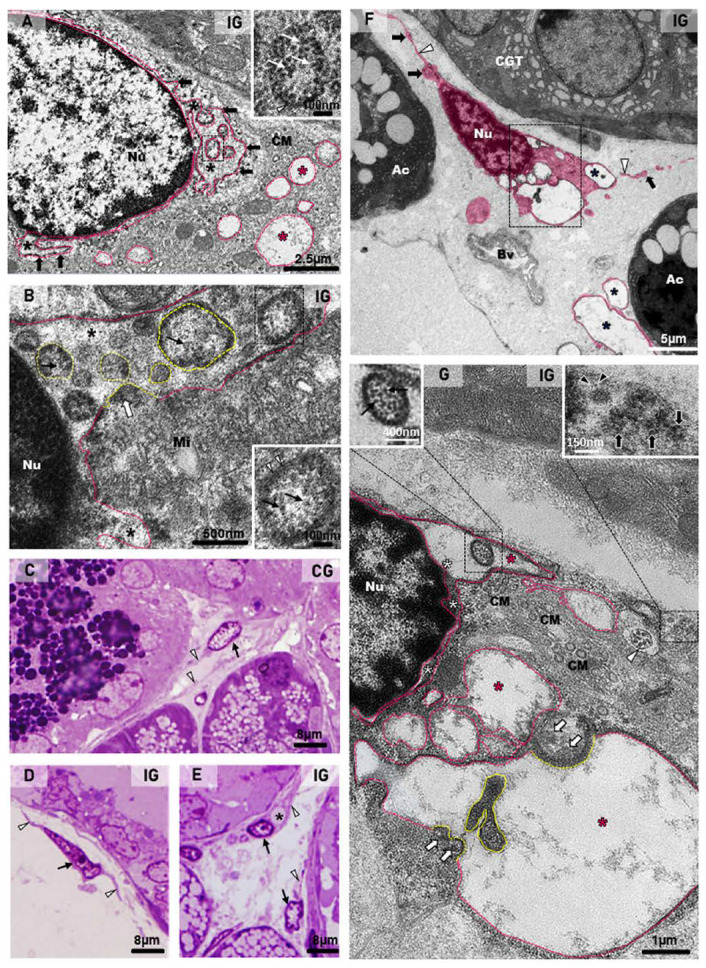
(**A**,**B**) Electron micrographs of portions of acinar cells of submandibular glands of animals from the IG. In (**A**), large vesicles (delimited by the pink line) are observed in the cytoplasm (pink asterisks). These vesicles are derived from dilations of the nuclear intermembrane space (black asterisks), forming irregular outlined vesicles (black arrows) continuous with the nuclear membrane. Some folded portions of these vesicles (named DMVs) are seen in cross sections within the dilation itself (black box and inset). Under high magnification (inset), a vesicle with double membrane (arrowhead) containing viral particles/nucleocapsid proteins (white arrows) is observed. Convoluted membranes (CMs) are also seen between the nuclear vesicles and the large cytoplasmic vesicles. In (**B**), a dilation of the nuclear intermembrane space (asterisks) forms a long vesicle (pink line) containing several DMVs (yellow lines) derived from the nuclear membrane folding itself, incorporating the cytoplasmic content with nucleocapsid proteins (white arrow), which are also observed inside DMVs (black arrows). Under high magnification (black box and inset), a DMV delimited by a double membrane (arrowheads) contains nucleocapsid proteins (black arrows). Mitochondria (Mi). Nu (nucleus). (**C**–**E**), Photomicrographs of semithin sections of SMGs stained by toluidine blue. In (**C**–**E**), telocytes with telopodes (arrowheads) are observed. In (**D**,**E**), the telocytes (arrows) show an atypical nucleus in comparison to (**C**). In (**E**), a vacuolar structure is observed in a telocyte cytoplasm (asterisk). (**F**,**G**) Electron micrographs of portions of SMGs of animals from the IG. In (**F**), a digitally colored electron micrograph shows portions of telocytes (pink) in the stroma. Note the moniliform aspect of the telopodes with an alternation between the podomers (white arrowheads) and the podoms (black arrows). Several vacuole-type vesicles are observed in the cytoplasm (asterisks). Bv (blood vessel); Ac (acini); GCT (granular convoluted tubule). In (**G**) (high magnification of (**F**)), a dilation of the nuclear intermembrane space (white asterisks) forming large vesicles (pink line and pink asterisks). In the nuclear vesicle, a DMV (black box) containing viral particles (left inset; black arrows) is observed. Note convoluted membranes (CMs) between the nucleus and the large vesicles. Nucleocapsid proteins (white arrows) are observed in viral assembly portions (yellow lines). A folded vesicle portion (delimited by the yellow line) inside the vesicle itself is observed. A vesicle containing viral particles (around 150 nm) is observed in the cell periphery (right inset; black arrows); in some of them, spike proteins (black arrowheads) can be seen. Note a centriole (white arrowhead) in the telocyte cytoplasm. Nu (nucleus).

**Figure 10:**
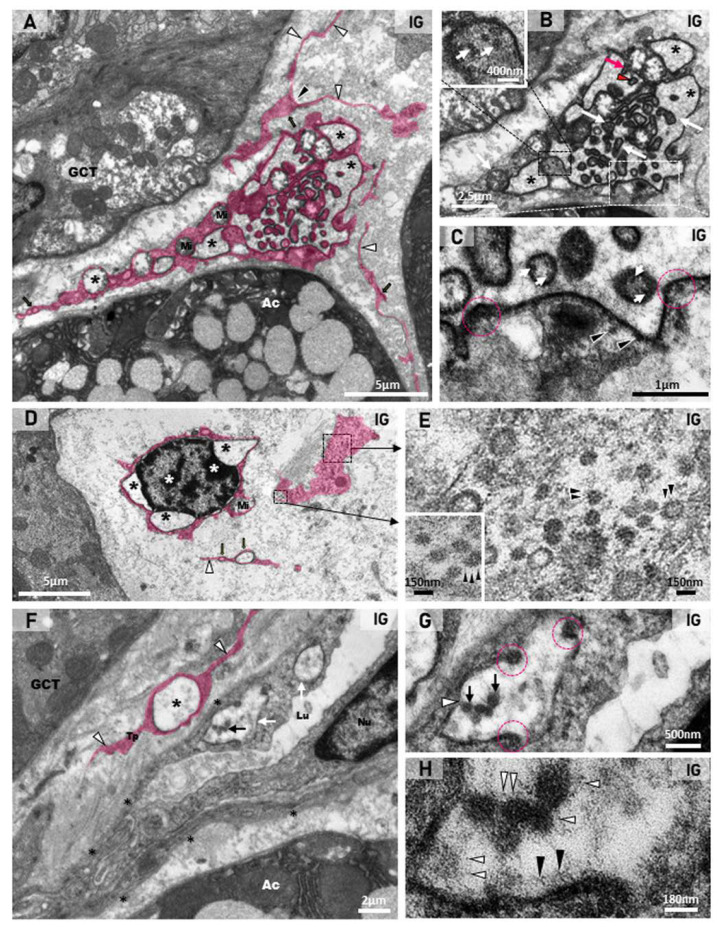
(**A**–**H**) Digitally colored electron micrographs of portions of stroma of SMGs of animals from the IG. In (**A**), a telocyte (pink) localized in the stroma between GCT and acinus (Ac). The telocyte shows a dichotomous pattern of branching (black arrowhead) and a moniliform aspect of the telopodes, with an alternation between the podomers (white arrowheads) and the podoms (black arrows). Note the large cytoplasmic portion containing convoluted vesicles (black asterisks). Mitochondria (Mi). In (**B**), a high magnification of (**A**), the convoluted large DMVs (asterisks) containing small, folded portions of the vesicles themselves (white arrows). Note the folded membrane during invagination (red arrow) and the formed vesicle after invagination (red arrowhead). In a large fold (black box and inset), note several viral particles, probably nucleocapsid proteins (white arrows). In (**C**) (high magnification of (**B**)—white box), several viral particles (nucleocapsid proteins) are seen outside the vesicle (arrowheads) and within the formed vesicles after invagination (white arrows). Note regions of the invagination process (pink circles). In (**D**), a dying telocyte with condensed clumps of chromatin in the nucleus (white asterisks) shows dilations of the nuclear intermembrane space (black asterisks). Mitochondria (Mi). In the cytoplasmic portion of the telocyte (black boxes), several viral particles are seen. In (**E**) (high magnifications of (**D**)—black boxes), note viral particles with spike proteins (arrowheads). In (**F**), a stroma portion between acinus (Ac) and GCT shows a collapsed blood vessel with a narrow lumen (Lu), surrounded by basal lamina (asterisks). Note DMVs (white arrows) with viral particles (black arrow) in the endotheliocyte. A DMV (white arrow) is also observed in a telopode (Tp) of a telocyte (in pink). Endotheliocyte nucleus (Nu). In (**G**,**H**) (high magnifications of DMV), viral particles (black arrows) and viral assembly sites containing nucleocapsid proteins (pink circles) are seen. In H, spike proteins are observed either on the viral surface (white arrowheads) or on the vesicle inner surface (black arrowheads).

**Figure 11:**
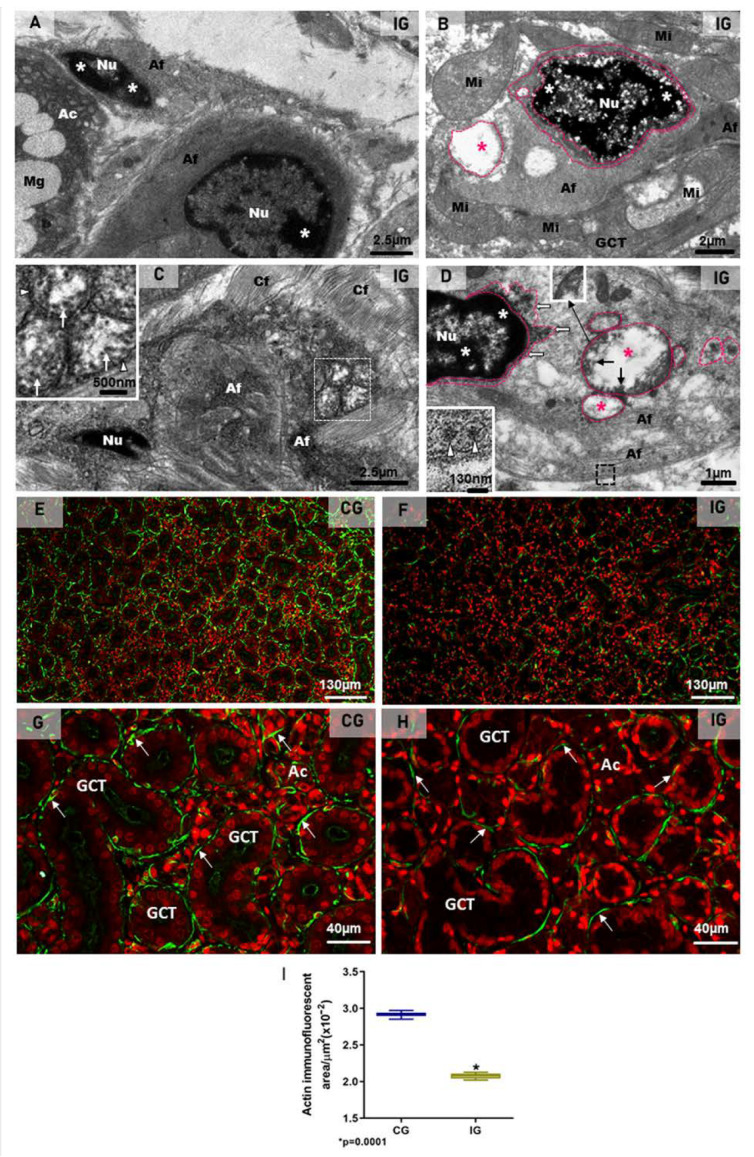
(**A**–**D**) Electron micrographs of the SMGs of animals from the IG showing portions of damaged MECs. In (**A**,**B**), dying MECs in close contact with acinus (Ac; (**A**)) and GCT (**B**) show electron dense masses of chromatin (asterisks) in the nucleus (Nu), and cytoplasm filled with actin filaments (Af). In (**B**), note the nuclear intermembrane space dilation (delimited by pink lines) and cytoplasmic vesicle (pink line and pink asterisk) derived from the nuclear dilation. Mucus granules (Mg); mitochondria (Mi). In (**C**), a portion of a damaged MEC shows cytoplasm filled with actin filaments (Af) and double membrane vesicles (white box and inset; white arrowheads). Several viral particles measuring around 130 nm are observed inside DMVs (inset; white arrows). In (**D**), a damaged MEC shows condensed chromatin (asterisks) in the nucleus (Nu). Note the DMVs (pink line and pink asterisks) derived from the nuclear intermembrane space dilation (pink line and white arrows). Double membrane of DMV is seen under high magnification (inset). Viral particles are observed either in groups, enclosed in large vesicles (black arrows), or isolated within small vesicles (black box and inset; white arrowheads) among actin filaments (Af), next to the plasma membrane. (**E**–**H**) Photomicrographs of SMGs showing actin immunofluorescence in MECs. In (**E**,**G**), strong and continuous actin immunolabelling (green; arrows) is observed in the MECs surrounding the acini and GCTs. In (**F**,**H**), a weak and discontinuous actin immunoreaction surrounding GCTs and acini (green; arrows) is observed in IG. In (**I**), a significant decrease in the actin immunofluorescent area is observed in IG in comparison with CG.

Dilations of the nuclear intermembrane space were also commonly found in different cell types. These dilations formed irregular outlined vesicles protruding towards the cytoplasm and were usually associated to large vesicles, normally delimited by a double membrane (DMVs), distributed throughout the cytoplasm (Figure 9A,B,F,G, Figure 10B–D,G and Figure 11D). In general, these vesicles showed a convoluted shape due to the invaginations of the membrane surface. Due to these invaginations, several small vesicle profiles (cross sections) were seen within the large dilations/vesicles themselves (Figure 9A,B,G and Figure 10B). Viral particles were found inside these vesicles (Figure 9A,B,G, Figure 10C,E–H and Figure 11C,D), and nucleocapsid proteins were also found in close contact with the vesicles’ outer membrane, indicating viral assembly (Figure 9B,F and Figure 10C,G). In the cell periphery, some small vesicles containing isolated viral particles measuring around 130 nm were also found (Figure 11D). In the infected cells, convoluted membranes (CMs) in cross and/or oblique sections were usually found between the nucleus and the large DMVs (Figure 9A,G). 

### 2.4. Viral Particles in Telocytes, MECs and Endotheliocytes

In the toluidine blue-stained semithin sections, telocytes showing scarce cytoplasm around the nucleus and long telopodes were usually found in the connective tissue stroma of the SMG in the animals from the CG (Figure 9C). However, in the IG, the telocytes showed an irregular nucleus and enlarged cytoplasm, in which vacuole-like structures were usually found (Figure 9D,E). Under TEM, telocytes exhibited typical features, such as scarce cytoplasm around the nucleus, long cytoplasmic projections, named telopodes, which showed a moniliform aspect, characterized by dilated cytoplasmic portions containing organelles (podoms) intercalated by thin cytoplasmic portions (podomers) (Figure 9F and Figure 10A,D,F). Occasionally, other typical features of telocytes, such as centrioles (Figure 9G) and a dichotomous branching pattern (Figure 10A), were also observed. In the IG, telocytes showed abnormal features, including a nucleus with condensed masses of chromatin, typical of cell death (Figure 10D), and enlarged cytoplasm containing DMV-like vesicles (Figure 9F,G and Figure 10A–C). The nuclear intermembrane space was also enlarged (Figure 9G and Figure 10D), forming large DMV-type vesicles. Within these vesicles, small ovoid or tubular vesicles (derived from the invagination of the DMVs themselves) were usually found (Figure 9G and Figure 10B). Nucleocapsid proteins were observed in close contact with the invaginating outer membrane of the DMVs (Figure 9G and Figure 10B,C), and also inside these vesicles (Figure 9G and Figure 10C). Moreover, convoluted membranes (CMs) between the nucleus and the large vesicles (DMVs) were also found in these damaged telocytes (Figure 9G). Some vesicles next to the plasma membrane showed assembled viral particles, in which spike proteins could be identified (Figure 9G and Figure 10E).

Some compacted stromal portions between the acini and the GCTs showed collapsed blood vessels with narrow lumen (Figure 10F). The endotheliocytes, surrounded by basal lamina, showed viral particles within the DMVs (Figure 10F–H). Evident spike proteins were observed either in the inner surface of the vesicles or surrounding the virus (Figure 10G,H). Next to the infected blood vessels, portions of telopodes of telocytes containing DMVs were found (Figure 10F).

Besides stromal cells (telocytes and endotheliocytes), DMVs and viral particles were also observed in the damaged myoepithelial cells (MECs), in close contact either with the acini or the GCTs. In these MECs, characterized by actin filaments-rich cytoplasm, the irregular nucleus showed clumps of condensed chromatin, typical of cell death (Figure 11A,B). Moreover, the dilation of the nuclear intermembrane space and large DMVs (derived from the dilation) were also observed in these cells (Figure 11B,D). Viral particles measuring around 70 to 150 nm were found either in clusters within large vesicles (DMVs), or isolated in small vesicles, next to the plasma membrane (Figure 11C,D).

The immunofluorescence for the detection of actin was observed surrounding the acini and GCTs in the SMG sections of the animals from the CG, indicating a typical and normal localization of MECs. However, in the IG, the immunoreaction was weak and discontinuous around the acini and GCTs (Figure 11E–H). Moreover, the area of actin immunofluorescence decreased significantly in the IG (Figure 11I).

## 3. Discussion

**Figure 12:**
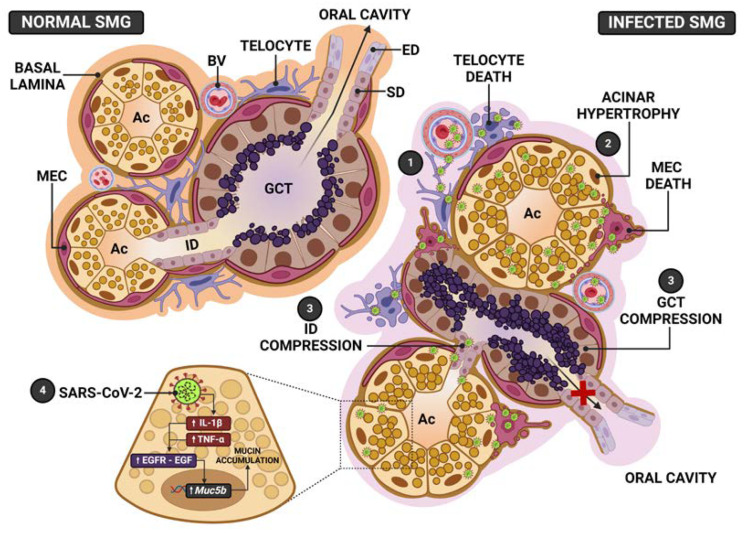
Schematic representation of the SMG pathogenesis following SARS-CoV-2 infection. In normal SMGs, the acini and GCT, showing normal distribution of the secretory granules, release their secretory content in the intercalated (ID) and striated (SD)/excretory ducts (ED), respectively, and transport saliva to the oral cavity. Myoepithelial cells (MECs) surround acini, GCT and ducts. In the stroma, telocytes interconnect blood vessels (BV), acini, ducts and GCT. In the infected SMG, (**1**) SARS-CoV-2 from the blood stream reaches telocytes and MECs. In these branched and supportive cells, the virus is replicated and transmitted to other cell types, allowing a rapid viral transmission, mainly to acinar cells. (**2**) In these cells, the infection induces mucin hypersecretion and accumulation of granules in the cytoplasm, culminating in the acinar hypertrophy. (**3**) The acinar enlargement causes ID and GCT compression, impairing the transport of saliva to the oral cavity. The loss of acinar structural support, due to telocytes and MECs death, favors the acinar enlargement caused by mucin hypersecretion. (**4**) SARS-CoV-2 infection triggers pro-inflammatory cytokines production (IL-1β and TNF-α), which activate EGF-EGFR signaling, causing *Muc5b* upregulation, culminating in the mucin hypersecretion and accumulation.

In the SARS-CoV-2-infected respiratory system, the virus stimulates mucin hypersecretion, causing a severe acute respiratory syndrome [38]. Considering that the salivary gland acinar cells are specialized in the production of mucin, which composes the saliva, we propose to evaluate the impact of SARS-CoV-2 infection on the SMG structural integrity, focusing on the mucin secretory activity by these cells. For this purpose, we used a K18-hACE2 transgenic mouse model, which expresses human ACE2 (hACE2), allowing the viral infection. Our results showed that SARS-CoV-2 intranasal inoculation was able to infect the SMG of K18-hACE2 mice, induced the production of pro-inflammatory cytokines by acinar and tubular cells, and caused structural and functional changes in this salivary gland. Interestingly, the infection caused a significant acinar hypertrophy, in contrast to GCTs atrophy. The acinar enlargement was due to mucin hypersecretion, confirmed by *Muc5b* overexpression. The intense EGF and cytokines immunoexpression, associated with *Egfr* upregulation, indicates a participation of the EGF-EGFR pathway associated to cytokines in the *Muc5b* upregulation (Figure 12).

The presence of dying myoepithelial cells (MECs) associated to actin reduction confirms that the structural framework provided by MECs to the acinar unit was impaired, favoring the acinar enlargement caused by mucus accumulation. The acinar hypertrophy compressed the GCTs, explaining tubular atrophy (Figure 12). These changes, associated with a loss of MECs contractility, explain the hyposalivation or xerostomia reported by COVID-19 patients [35,36]. The presence of telocytes and MECs in cell death, showing viral assembly, points to these intercommunicating cells as viral targets that favor SARS-CoV-2 replication and cell-to-cell transmission. 

### 3.1. Submandibular Gland Cells Express hACE2 and Are Infected by SARS-CoV-2

ACE2 and TMPRSS2 proteins have been detected by immunoreactions in the acinar and ductal cells of the parotid, SMGs, sublingual glands and minor salivary glands [4,39,40]. Moreover, SARS-CoV-2 RNA, as well as viral particles, have also been detected in the postmortem biopsies of the SMGs and parotid glands of COVID-19 patients [3]. SARS-CoV-2 infection has also been demonstrated in the acini and ducts of human minor salivary glands [4] and SMGs [41]. Our findings corroborate these studies, since either the acinar or the tubular cells of the SMGs in K18-hACE2 transgenic mice were positive for hACE2. Moreover, spike and nucleocapsid immunolabelling was detected in several SMG cells of the infected animals, mainly acinar cells.

The analyses under TEM were undoubtedly essential for the identification and subcellular localization of nucleocapsid proteins and viral particles in SMG cell types, as well as for the understanding of the pathogenesis induced by the viral infection. Among in situ detection techniques, TEM is essential to identify assembled viruses and confirm SARS-CoV-2 replication in the cells. We considered the main criteria to identify the viral particles, such as size (60 to 150 nm), the presence of small electron-dense dots, corresponding to the helical nucleocapsid, which may be surrounded by an envelope in case of an assembled virus [42]. Moreover, virus assembly, characterized by the budding of the nucleocapsid proteins into the membrane-limited vesicles [42], was also considered. In our study, the ultrathin sections were conventionally stained; thus, the identification of spike proteins, which are normally identified in tannic acid-stained sections, was not readily discernible, except for a few images (depending on the section plane), in which spike proteins could be identified [42,43]. However, in the present study, the immunofluorescence reaction for the detection of spike proteins confirmed the presence of this viral protein in the SMG acinar cells, and a strong nucleocapsid protein immunoreaction was also detected in the acinar cells. Besides the virus, other ultrastructural features were taken into account to confirm SARS-CoV-2 infection, such as the presence of CM and DMVs, or single-membrane vesicles [42]. In the SMG cells of the animals from the IG, viral components (nucleocapsid proteins) and/or viral particles (assembled viruses), as well as virus assembly, were observed in association with, or independently of CM and DMVs. MECs, telocytes in advanced stages of cell death and endotheliocytes showed clusters of viral particles inside large vesicles and/or single viruses inside small vesicles next to the plasma membrane. Free viral particles were also found in the lumen of ductal cells. Therefore, these findings confirm that SARS-CoV-2 shows a tropism for the SMG cells, pointing to these cells as replication factories and reservoirs of SARS-CoV-2, corroborating the potential airborne transmission of SARS-CoV-2-rich salivary droplets [1].

### 3.2. A Possible Role of the Nucleus in Viral Replication

The viral entry into the host cell induces intracellular membrane remodeling, which gives rise to the replication membranous web (RMW), a three-dimensional vesicular structure that indicates viral replication. This structure is interconnected with single-membrane vesicles (CMs) and DMVs. Virus assembly is characterized by the budding of the nucleocapsid proteins into the cell membranes, and the assembled SARS-CoV-2 viruses are normally found inside vacuoles/vesicles [42]. In SARS-CoV-2-infected Vero cells, studies have demonstrated DMVs in continuity with the endoplasmic reticulum (ER), suggesting that these vesicles are derived from the ER membrane. Moreover, the Golgi apparatus has also been correlated to DMVs formed by other viruses, indicating a remodeling of both ER and Golgi apparatus membranes [44]. In the present study, our findings strongly point to a participation of the nuclear membrane in the formation of viral replication vesicles, since most of the infected glandular cells showed dilations between the inner and outer nuclear membranes (i.e., nuclear intermembrane space), forming vesicular protrusions towards the cytoplasm. The convoluted folding of these dilations in cross section showed several small vesicles inside the dilations themselves. Since the RER membrane is continuous with the nuclear membrane, we hypothesize that the formation of the outer nuclear membrane protrusions towards the juxtanuclear RER cisternae causes points of fusion between the nuclear membrane and the RER membrane, and the subsequent incorporation of these membranes into each other, giving rise to a unique, dilated and long vesicle derived from the nuclear membrane + ER membrane (NER), resulting in convoluted and narrow NER vesicles in the perinuclear region extending towards the cytoplasm. These NER vesicles and DMVs (probably large portions of NER) could favor the transport of viral replication proteins from the nucleus to these supposed replication vesicles. These findings are reinforced by the evident immunolabelling of nucleocapsid proteins observed in the nuclear periphery of the infected cells. In fact, corroborating our findings, a study showed the SARS-CoV-2 nucleocapsid protein in the perinuclear area colocalized with the spike protein and a Golgi marker antibody by immunofluorescence [45]. Moreover, a recent study has demonstrated that after SARS-CoV-2 enters the cell, viral RNA-derived polypeptides form replication and transcription complexes (RTCs), whose subunits (Nsp3, Nsp4 and Nsp6) play a role in the formation of CMs and DMVs, named replication organelles. These organelles, located in the perinuclear region, were interconnected and connected to the ER, providing communication between the nuclear lumen and the DMVs’ intermembrane space [46]. Therefore, these findings corroborate our ultrastructural images, which show, for the first time to our knowledge, dilations of the nuclear intermembrane space, forming protrusions and vesicles towards the cytoplasm of SAR-CoV-2-infected cells. Undoubtedly, these protrusions may fuse to the RER and Golgi complex, giving rise (possibly) to DMVs. These findings reinforce the participation of the nucleus in the formation of viral replication vesicles/organelles.

### 3.3. Viral Infection Induces Cytokine Production in Acinar Cells and Mucin Hypersecretion

Studies have demonstrated that the acinar cells under normal conditions synthesize, store and release IL-1β and IL-6 in the mouse parotid [47]. Moreover, IL-1α and IL-1β have been detected in the acinar and ductal cells of the minor salivary glands of patients with SS and sialoadenitis [48]. In the current study, an intense immunoexpression of IL-1β and TNF-α was also observed in the acinar and GCT cells of the infected SMGs, confirming a specific “inflammatory response” of these epithelial cells to the viral infection. The mucin hypersecretion induced by SARS-CoV-2 leads to mucin accumulation in the respiratory tract and the obstruction of the airway, impairing breathing and recovery. This process results in the development of the acute respiratory distress syndrome (ARDS) in the COVID-19 patients, and it is regulated by cytokines, such as IL-1β and TNF-α [49]. A few studies have reported significant upregulation of several mucin genes, including the gel-forming mucins such as MUC5AC and MUC5B by the bronchiolar epithelial cells of COVID-19 patients [50]. In some autopsies, severe mucoid tracheitis was detected, confirming that the respiratory distress is caused by mucin hypersecretion [33]. Moreover, respiratory mucus accumulation has been associated with *Muc5b* overexpression in the autopsied lungs of COVID-19 patients [28]. Therefore, we hypothesize that the process involving the intense cytokine immunoexpression associated with mucin hypersecretion in the acinar cells of the infected SMGs is the same process as that described in the goblet cells of the respiratory tract of individuals with COVID-19. This hypothesis is reinforced by the intense EGF immunoexpression detected in GCTs associated with the *Egfr* upregulation in the infected SMGs. The overexpression of *Egfr* and its ligand (EGF) is involved in mucin hypersecretion-induced diseases. The mucin production in the respiratory tract cells during airway diseases is stimulated by the EGFR signaling [30,51]. EGF also increases the number of goblet cells, as well as the AB-PAS-positive mucin and the expression of the *Muc5ac* gene [30], whose overexpression is mediated by EGF signaling [31]. Moreover, MUC5B-rich mucus hypersecretion induced by SARS-CoV-2 in human bronchial epithelial cultures seems to be mediated by EGFR and IL-1R-dependent pathways [28]. TNF-α stimulates *Egfr* expression, which binds to its ligand EGF and induces *Muc5ac* gene expression, stimulating mucus hypersecretion in airway epithelial cells [33,52]. The TNF-α/EGF-EGFR-induced MUC5AC production was confirmed by studies using EGFR inhibitors, which block the *Muc5ac* expression [30]. Moreover, the non-secretory cells of the airway tract are differentiated from mucin-secreting goblet cells when TNF-α induces EGFR, confirming that EGFR-EGF mediates mucin secretion under TNF-α control [30]. In the present study, a significant increase in EGF, IL-1β and TNF-α immunolabelling was associated to *Egfr* upregulation in the infected SMGs. Since EGFR has been detected in both the acinar and ductal cells of the salivary glands [53], the mucin hypersecretion by the acinar cells observed in the SARS-CoV-2-infected SMGs is mediated by the EGF-EGFR signaling pathway, and cytokines seem to mediate this process (Figure 12).

### 3.4. SARS-CoV-2 Induces MECs Death and Acinar Hypertrophy: A Sialadenosis-like Feature

Intriguingly, the mucin hypersecretion by the acinar cells observed here is not supported by the common effects in the oral cavity, reported by COVID-19 patients, such as salivary glands dysfunction and xerostomia [35,36,54]. Similar effects have also been associated with salivary gland ectasia [55], parotitis [56], sialadenitis [4,57], sialadenosis [19,58], as well as Sjögren’s syndrome (SS) [59,60]. A study has demonstrated that the viral infection seems to induce an SS-like disease either in SARS-CoV-2-infected mice or in COVID-19 patients [59]. The main histopathological features of this disease (SS) [60], such as acinar hypertrophy and acinar cells filled with mucus granules and dilated RER cisternae, are similar to those observed in the current study. The mucin hypersecretion induced by this disease has also been related to NF-kB activation and the synthesis of pro-inflammatory cytokines [60]. Therefore, the pathogenesis induced by SARS-CoV-2 in the SMGs is similar to that induced by cytokines in the salivary glands affected by SS.

In the present study, the presence of dying MECs, associated with actin reduction, confirms that these cells were impaired in the infected SMGs, a damage that may explain hyposalivation/xerostomia in COVID-19 patients. Xerostomia has been associated with disturbances in the sympathetic and/or parasympathetic nervous stimuli, which normally act directly on the MECs, whose long processes surround and embrace the acini and ducts [17]. In response to stimuli, the MECs myofilaments contract, favoring the mechanical compression of the acini and ducts, allowing degranulation and release of saliva through the ductal system [17]. After contraction, MECs stabilize the acinar structure [61], maintaining the mechanical support of the acinar unit [19]. Thus, when myofilaments and or MECs are disrupted, the glandular excretory function is impaired, and the secretory granules are accumulated in the cytoplasm, leading to acinar cell enlargement [19]. This specific disturbance also occurs in sialadenosis, a pathological process in which the acinar cells are hypertrophied due to an impaired secretory process, and the main cause has been related to MECs’ atrophy due to autonomic nervous system dysfunction [19,58]. A significant reduction in the number of MECs and myofilaments, as well as the dysfunction of these cells, results in the reduction in saliva flow and xerostomia in sialadenosis [19]. In the current study, the histopathological features observed in the infected SMGs were similar to those observed in sialadenosis, indicating that the infection of MECs by SARS-CoV-2 and subsequent death may have favored the acinar hypertrophy (Figure 12). Although neural structures were not analyzed, we cannot omit the possibility that MECs’ atrophy could be induced by neural dysfunction due to infection. However, either spike immunolabelling or SARS-CoV-2 particles were detected in the MECs of the IG. As these stellate cells contain several long cytoplasmic processes that embrace the acini on one side and are in close proximity to the stroma and blood vessels on the other side, it is conceivable to suppose that the large surface of MECs renders these cells susceptible to viral infection and favors viral transmission to the acinar unit. Moreover, actin filaments are strictly involved in viral function, infectivity and pathogenicity [62,63], since these filaments play essential roles in viral internalization, replication, transport through the cell and viral particle egress. Therefore, MECs may be potential targets of SARS-CoV-2 in the infected salivary glands, favoring a rapid viral transmission to the acinar cells.

It is important to emphasize that the acinar hypertrophy, observed in the IG, compressed the GCTs, whose diameter and Vv reduced significantly (Figure 12). This compression was confirmed by the presence of atrophic GCT portions and clusters of GCT cells enclosed by large AB-positive acini, as well as the absence of a basement membrane in the acini-GCTs interface, confirming the interconnection and fusion between these structures. In mice, the intercalated ducts and GCTs (Figure 12) allow the passage of saliva from the acinar units and intercalated ducts to the striated ducts [11]; therefore, the hyposalivation reported by COVID-19 patients might be a consequence of MECs’ contractile dysfunction associated with the obstruction of the ductal system caused by acinar hypertrophy, impairing the salivary flow to the oral cavity (Figure 12).

### 3.5. Telocyte as Possible SARS-CoV-2 Target and Viral Transmitter in SMGs

In the present study, the telocytes showed their typical long processes (telopodes), with thin portions (podomers) intercalated with dilated portions (podoms), confirming its moniliform feature. Moreover, some telocytes showed a dichotomous branching pattern and centriole, which are typical features of this cell type [20]. The long telopodes, in contact with each other and with other cell types, form an intricate network, providing a supportive framework in the organs [27]. In the lungs, damage to telocytes is associated with a lack of architectural integrity, homeostasis and dysfunction of these organs. Most telocytes found in the infected SMGs showed abnormal features, such as electron-dense chromatin in the nuclear periphery, typical of cell death, dilations of the nuclear intermembrane space and convoluted vesicles containing viral particles, confirming SARS-CoV-2 infection in these cells. However, whether the death of the telocytes in the infected SMGs is the cause or the consequence of acinar hypertrophy needs to be clarified. In some pathological conditions such as salivary gland in SS [25] and inflammatory bowel disease, changes in the structure, homeostasis and/or function of these organs have been correlated with disturbances in telocytes [26].

The cytoplasmic processes of telocytes connect acinar, tubular/ductal cells and capillaries. Thus, as the virus can reach the salivary glands via the blood stream, it is conceivable to suggest that telocytes may be the first cells infected by the virus (Figure 12). In fact, our findings showed telopodes next to infected endotheliocytes. Due to the intricate network that these cells establish through contacts between themselves and with other cell types, once infected, these cells may readily transmit viral particles to all other glandular cell types. This could explain why the salivary glands are susceptible to the infection and become intensely infected, storing a high viral load, which is detected in saliva before pulmonary manifestations in the asymptomatic COVID-19 patients.

## 4. Conclusions

The SMG cells of K-18hACE2 transgenic mice express hACE2 and, after five days of SARS-CoV-2 infection, showed spike and nucleocapsid proteins and/or viral particles in the acinar cells, GCT cells, ductal cells, myoepithelial cells, telocytes and endothelial cells. The dilations of the nuclear intermembrane space associated to the presence of viral particles point to a potential role of the nucleus in the viral replication and formation of DMVs. Therefore, the K-18hACE2 transgenic mouse is a useful model for the investigation of SARS-CoV-2 infection mechanisms in different SMG cell types. The intense acinar hypertrophy and mucin accumulation was due to *Muc5b* overexpression via EGF-EGFR signaling. Either the loss of myoepithelial cells, impairing acinar/ductal contraction, or the ductal obstruction by acinar hypertrophy corroborates hyposalivation/xerostomia in COVID-19 patients. Since salivary gland hypofunction may disturb pH conditions and impairs the oral microbiota, attention is necessary to the oral health of infected individuals regarding short and long-term effects of COVID-19.

The acinar cell capacity of mucin synthesis, associated with the structural features of telocytes and myoepithelial cells, seems to favor the viral replication and transmission in the infected SMG, making this organ susceptible to viral infection and replication. These findings may explain the high viral load in saliva and the prompt transmission by asymptomatic COVID-19 individuals.

## 5. Material and Methods

### 5.1. Preparation of SARS-CoV-2 Samples

SARS-CoV-2 was isolated from a COVID-19 positive-tested patient. The virus was propagated and titrated in Vero E6 cells in a biosafety level 3 laboratory (BSL3) at Ribeirão Preto Medical School (University of São Paulo-USP, Ribeirão Preto, Brazil). Cells were cultured in DMEM medium supplemented with 10% fetal bovine serum (FBS) and antibiotic/antimycotic drugs (penicillin 10,000 U/mL; streptomycin 10,000 μg/mL). The viral inoculum was added to Vero cells in DMEM (FBS 2%) incubated at 37 °C with 5% CO_2_ for 48 h, and the cytopathogenic effect was observed under microscope. The cell monolayer was collected, and the supernatant was stored at −70 °C. Virus titration was made by plaque-forming units (PFU).

### 5.2. K18-hACE2 Mice and SARS-CoV-2 Infection

K18-hACE2 mice were obtained from the Jackson Laboratory and were maintained in a biosafety level 3 (BSL3) facility at Ribeirão Preto Medical School/University of São Paulo (USP, Brazil). K18-hACE2 transgenic mice have been used as a model for SARS-CoV-2-induced disease since the clinical signs and the biochemical and histopathological changes observed in the infected animals are compatible with the human disease [64]. Mice were maintained in adequate cages with water and food ad libitum and standardized environmental conditions, such as photoperiod (12 h light/12 h dark), temperature (23 ± 2 °C) and humidity (55 ± 10%).

Twelve K18-hACE2 mice, aged 8 weeks, were distributed into two groups: CG (control; n = 6) and IG (infected; n = 6). The animals from the IG were infected with 5 × 10^4^ PFU of SARS-CoV-2 (in 40 µL) by intranasal route. Control mice were inoculated with an equal volume of DMEM. The protocol regarding the use and treatment of the animals was approved by the Ethical Committee of Ribeirão Preto Medical School-USP (protocol number 021/2021) and Araraquara Dental School-UNESP (protocol number 03/2022).

### 5.3. Histological Procedures for Light Microscope

After treatment, the animals were weighed and anaesthetized with 80 mg/kg BW of ketamine hydrochloride (Francotar, Virbac do Brasil Ind. Com. Ltd.a, Jurubatuba, Brazil, Reg.MA: 7.885) and 8 mg/kg BW of xylazine hydrochloride (Virbaxyl; Virbac do Brasil Ind. Com. Jurubatuba, Brazil, Reg. MA: 7.899).

The SMGs were fixed for 48 h in 4% formaldehyde (Merck, Germany) buffered with 0.1M sodium phosphate (pH 7.4). The samples were dehydrated in graded concentrations of ethanol and embedded in glycol methacrylate (Leica Biosystems, Historesin-Embedding Kit, Wetzlar, Germany, lot: 010284) or paraffin. The historesin sections (3 µm thick) were stained with Gill’s Hematoxylin and Eosin (HE), according to Cerri and Sasso-Cerri [65] for morphological and morphometric analyses. The paraffin sections were submitted to the Periodic-Acid-Schiff (PAS) method, Alcian Blue (AB) histochemical method, silver impregnation, TUNEL (TdT-mediated dUTP-biotin Nick End Labeling) method, immunohistochemistry and immunofluorescence reactions, as described below.

### 5.4. Morphometric Analyses

For the morphometric analyses, the images were captured using a camera (DP-71; Olympus, Tokyo, Japan) attached to a light microscope (BX-51; Olympus), and an image analysis system (Image Pro-Express 6.0; Olympus) was used.

### 5.5. Volume Density of Acini and Ducts

In four HE-stained submandibular gland non-serial sections (the distance between sections was around 30 µm), twelve fields of glandular tissue per animal were randomly selected. A 240-point grid, created by the Image Analysis System–Image Pro-Express 6.0 (Olympus, Tokyo, Japan), was superimposed on the glandular tissue, the number of points on each component (acini and GCTs) was counted, and the volume density (Vv) of these components (acinar Vv and GCT Vv) was calculated [66].

### 5.6. GCTs Diameter and Area of PAS^+^ Secretion

In HE-stained non-serial sections, twelve fields of glandular tissue per animal were captured. In each field, the tubular minor axis was measured in 20 GCTs, totaling 240 tubules per animal.

Sections were stained with Periodic acid-Schiff reagent according to Cerri and Sasso-Cerri [65]. The sections were immersed in 1% periodic acid at room temperature, washed and immersed in Schiff’s reagent for 30 min. After washings, the sections were dehydrated and mounted in Permount.

In PAS-stained sections, the area of PAS^+^ secretion was measured in a standardized tubular area of around 100,000 µm^2^ (around 20 GCTs per animal), and the area of PAS^+^ secretion per µm² of GCT was calculated.

### 5.7. Histochemical Methods: AB and Silver Impregnation

For the detection of glycoconjugates containing acid radicals (mucin), the SMG sections were immersed in 0.1N HCl (pH 1.0) for 5 sec, and in a solution containing 1% Alcian Blue (AB) in HCl 0.1N, for 30 min. After washes in HCl 0.1N, the sections were washed in dH_2_O, counterstained with Harris’ hematoxylin, dehydrated and mounted in a resinous medium [65].

The method of silver impregnation was performed for the identification of the basement membrane. The deparaffinized and hydrated sections were incubated in 1% potassium permanganate (1 min), washed in distilled water (dH_2_O) and immersed in 3% oxalic acid (3 min). After washings in dH_2_O, the sections were immersed in 2% iron alum (1 min), washed, and immersed in an ammoniacal silver nitrate solution for 1 min. After washings in dH_2_O, the sections were fixed in 10% formaldehyde, immersed in 1% gold chloride (10 min), washed in dH_2_O and immersed in 5% sodium thiosulfate (1 min). The sections were washed, stained by hematoxylin, dehydrated and mounted with a resinous medium.

### 5.8. Immunofluorescence Reactions

In order to confirm the presence of the human angiotensin-converting enzyme 2 (hACE2) in the glandular sections of K18-hACE2 mice, as well as the viral presence of SARS-CoV-2 in the glands of the infected animals, immunofluorescence reactions for the detection of hACE2, spike protein and nucleocapsid protein were performed. Moreover, actin, EGF (epidermal growth factor) and TNF-α (pro-inflammatory cytokine) were also detected by immunofluorescence.

Non-serial paraffin sections were immersed in 0.001 M citrate buffer (pH 6.0) and heated in a microwave oven at 90 °C for 30 min for antigen recovery. All sections were incubated in 2% BSA for 30 min and incubated at 4 °C overnight with the following primary antibodies: mouse anti-ACE2 (AC18Z) monoclonal antibody (1:500, RRID: AB_1118590, Santa Cruz Biotechnology; code: sc-73668); rabbit anti-SARS-CoV-2 spike protein S1 monoclonal antibody (1:500; RRID: AB_2890589, Thermo Fisher Scientific–Waltham, MA, USA, code: MA5-36247); rabbit anti-SARS-CoV-2 nucleocapsid protein monoclonal antibody [1:3000; EPR24334-118; Abcam, Cambridge, MA, USA; ab271180]; rabbit anti-actin polyclonal antibody (1:1000, RRID: AB_476693; Sigma-Aldrich, Germany; code: A2066); rabbit anti-EGF polyclonal antibody (1:300; RRID: AB_2877636, Boster Biological Technology, Pleasanton, CA, USA, code: A00378-1); and mouse anti-TNF-α monoclonal antibody (1:500, RRID: AB_302615, Abcam, Cambridge, MA, USA; code: ab1793). After washings in PBS, the sections were incubated in the dark at room temperature, with the following secondary antibodies: Alexa Fluor^®^488 anti-mouse IgG antibody (1:1000; Molecular Probes^®^ by Life Technologies, Carlsbad, CA, USA, A11001, lot: 1664729) and Alexa Fluor^®^594 anti-rabbit IgG antibody (1:500; Invitrogen^®^ by Thermo Fisher Scientific, Carlsbad, CA, USA, code: R3117, lot: 2086924) for 1 h at room temperature. After washings in PBS, nuclear staining was performed with DAPI (1:500, Molecular Probes by Life Technologies; Carlsbad, CA, USA, R37606, lot:1616913) for 5 min in the dark at the room temperature, and the slides were mounted with a Fluoromount^®^ mounting medium (Dako Faramount Aqueous mounting medium, Dako Inc., Carpinteria, CA, USA, S3025, lot: 11176284). To check possible unspecific binding of the secondary antibodies to the tissues, negative controls were performed by incubating sections with non-immune serum instead of primary antibodies.

### 5.9. Spike and Actin Double Immunofluorescence

After antigen recovery, as described above, the sections were incubated with a rabbit anti-SARS-CoV-2 spike protein S1 monoclonal antibody (1:500, RRID: AB_2890589, Thermo Fisher Scientific–USA, code: MA5-36247) overnight, at 4 °C. After washings in high salt PBS, the sections were incubated with an Alexa Fluor^®^594 anti-rabbit antibody (1:1000; Invitrogen^®^ by Thermo Fisher Scientific, Carlsbad, USA, code: R3117, lot: 2086924) for 1 h at room temperature. The following day (second day), the sections were incubated with a rabbit anti-actin polyclonal antibody (1:200, RRID: AB 476693; Sigma-Aldrich, Germany; code: A2066) overnight, at 4 °C. The following day (third day), the sections were washed in high salt PBS and incubated with an Alexa Fluor^®^488 anti-rabbit IgG antibody (1:1000; Molecular Probes^®^ by Life Technologies, Carlsbad, USA, A11001, lot: 1664729), for 1 h, at room temperature. After washings in high salt PBS, nuclear staining was performed with DAPI (1:500, Molecular Probes by Life Technologies; Carlsbad, CA, USA, R37606, lot:1616913) for 5 min, in the dark, at room temperature, and the slides were mounted with a Fluoromount^®^ Mounting Medium (Dako Faramount Aqueous Mounting Medium, Dako Inc., Carpinteria, CA, USA, S3025, lot: 11176284). Negative controls were performed following the same protocol and steps, except for the incubations in primary antibodies, which were replaced by non-immune serum.

The immunofluorescence was analyzed using a DFC 550 camera (Leica, Wetzlar, Germany) attached to a BM4000 B LED microscope (Leica, Germany) and the image analysis system—Leica Application Suite software (LAS 4.3, Leica, Germany).

### 5.10. Analysis of Immunofluorescent Areas

Immunofluorescent areas were analyzed using a DFC 550 Camera (Leica, Germany) attached to a BM4000 B LED microscope (Leica, Germany). An image analysis system— Leica Application Suite software (LAS 4.3, Leica, Germany)—was used for the measurement of areas. All parameters of the software, including exposure, gain and saturation, as well as the threshold adjustment and color range, were rigorously standardized for each immunoreaction and analyzed so that only areas with intense red or green fluorescence were computed.

For the measurement of actin immunoreaction, non-serial glandular sections from each animal were used. From these sections, 20 fields of glandular tissue were randomly captured at ×40, totaling a standardized glandular area of around 300,000 µm^2^ per animal. In this area, the immunofluorescence of actin was measured, and the actin immunofluorescent area per µm^2^ of glandular tissue was calculated.

To estimate the immunofluorescent area of EGF, non-serial glandular sections were used. From these sections, five fields were captured at ×40, totaling 20 fields per animal. The area of GCTs was measured, totaling a standardized GCT area per animal. In this area, the EGF immunofluorescence was measured, and the EGF immunofluorescent area per µm^2^ of GCT was calculated.

### 5.11. Transmission Electron Microscopy (TEM)

Fragments of SMGs were processed for TEM according to Beltrame et al. [67]. Specimens were immersed in a solution of 4% glutaraldehyde and 4% formaldehyde buffered at pH 7.2 with 0.1 M sodium cacodylate at room temperature for 17h. Subsequently, the specimens were post-fixed in 1% osmium tetroxide buffered in 0.1 M sodium cacodylate (pH 7.2) for 1 h, washed in dH_2_O and immersed for 2 h in 2% aqueous uranyl acetate at room temperature. After washing in dH_2_O, the specimens were dehydrated in graded concentrations of ethanol, treated with propylene oxide, and then embedded in Araldite. Semithin sections stained with the aqueous solution that contained 1% toluidine blue and 1% sodium borate were examined under a light microscope, and suitable regions were carefully selected for trimming the blocks. Ultrathin sections were collected on cooper grids, stained in alcoholic 2% uranyl acetate and in a lead citrate solution and examined using an FEI transmission electron microscope (TECNAI model).

### 5.12. Reverse Transcription and Real-Time Polymerase Chain Reaction (RT-qPCR)

Immediately after collection, the SMG fragments were immersed in an RNA Keeper stabilizing reagent (LGC Biotecnologia; Cotia, Brazil; 14-0002-01) and stored at 80 °C. For RNA extraction, the Aurum Total RNA Mini Kit (Bio-Rad, Hercules, CA, USA; 732-6820) was used, and reverse transcription was performed using the High-Capacity cDNA Reverse Transcription Kit (Applied Biosystems, Cheshire, UK; 4368814). The primers used for RT-qPCR reactions are listed in Table 1. The reactions were performed using the Power Up SYBR Green Master Mix (Applied Biosystems, Cheshire, UK; A25742) and a QuantStudio 3 Real-Time PCR instrument (Applied Biosystems, Cheshire, UK). The thermal cycling conditions were applied according to the Power Up SYBR Green Master Mix protocol. For gene expression analysis, the results were reported as mean  ±  SD, using the formula ΔCt  =  [Ct target gene Ct housekeeping gene β-actin]. Relative expression is derived from log(2^−ΔΔCt^), where ΔΔCt  =  ΔCt of the SMG of IG—the mean of ΔCt from the control group. Primer design was performed using the murine sequences available at the University of California, Santa Cruz (UCSC) Genome Browser and the Primer3 program [68].

### 5.13. Statistical Analysis

The statistical analysis of the data was performed using GraphPad Prism^®^ 8.4.3 software (GraphPad Software, La Jolla, CA, USA). The data were checked for normal distribution by the Kolmogorov and Smirnov’s normality test. According to the data distribution, the differences between groups were evaluated by Student’s *t*-test. The accepted significance level was *p* ≤ 0.05.

## Figures and Tables

**Table 1:** Sequence of primers used in qPCR.

Gene	NCBI Access No:	Length (bp)	Oligonucleotides Sequences (5′-3′)	Tm
*Muc5b* (Exxtend, Brazil)	NM_028801.2	20 20	F: GTGTCGGGCAGAGAACTACC R: GGGCTTGTCTACTCACCAGG	60.0° 60.0°
*Egfr* (Exxtend, Brazil)	NM_207655.2	20 20	F: TTTAGTCCCTCCTCCTCCCG R: CTAGCCCAGTCTCCCTTCCT	60.0° 60.0°
*β-Actin* (Exxtend, Brazil)	NM_007393.5	18 20	F: CTGCGCTTCCTTTGTCCC R: GACAATTGAGAAAGGGCGTG	57.0° 55.0°

## Data Availability

The data presented in the study are available upon request from the corresponding authors.
